# Tizanidine Toxicity From Ciprofloxacin: A Cautionary Tale

**DOI:** 10.7759/cureus.32492

**Published:** 2022-12-13

**Authors:** Lokesh Goyal, Deobrat Mallick, Miana R Zapata, Kanica Yashi, Prabal Chourasia, Salim Surani

**Affiliations:** 1 Hospital Medicine, Christus Spohn Hospital Corpus Christi - Shoreline, Corpus Christi, USA; 2 Internal Medicine, Christus Spohn Hospital Corpus Christi - Shoreline, Corpus Christi, USA; 3 Internal Medicine, University of the Incarnate Word School of Osteopathic Medicine, San Antonio, USA; 4 Internal Medicine, Bassett Health Care, Cooperstown, USA; 5 Hospital Medicine, Mary Washington Hospital, Fredericksburg, USA; 6 Anesthesiology, Mayo Clinic, Rochester, USA; 7 Medicine, Texas A&M (Agricultural and Mechanical) University, College Station, USA; 8 Medicine, University of North Texas, Dallas, USA; 9 Internal Medicine, Pulmonary Associates, Corpus Christi, USA; 10 Clinical Medicine, University of Houston, Houston, USA

**Keywords:** p450, cyp1a2, cytochrome p450 cyp1a2, ciprofloxacin, hypotension, muscle spasticity, alpha-2 adrenergic agonist, tizanidine

## Abstract

Tizanidine is an alpha-2 adrenergic agonist used commonly by medical professionals to treat patients' chronic spasticity, muscle spasms, and neuralgia usually associated with myofascial components. This medication is also used very frequently in detoxification centers on patients treated for analgesic withdrawal, especially those who are suffering from rebound headaches due to the discontinuation of analgesics. Tizanidine is metabolized in the human body by the cytochrome P450 CYP1A2. On the other hand, ciprofloxacin is a common antibiotic belonging to the class of fluoroquinolones and is used to treat various infections. Ciprofloxacin inhibits the bacterial DNA-gyrase enzyme resulting in the destruction of the organism. Ciprofloxacin is also an inhibitor of the cytochrome P450 CYP1A2. Even though these two medications show obvious interaction still, however, both these medications are often prescribed together, and their interactions/contraindications are often overlooked by many physicians and other providers. We hereby describe the case report of the interaction between tizanidine and ciprofloxacin, along with the adverse outcome related to the concomitant use of these two drugs.

## Introduction

Tizanidine is an alpha-2 adrenergic agonist used to treat moderate-to-severe muscle spasms usually caused by diseases like multiple sclerosis, spinal cord injuries, and various other medical conditions. Cytochrome P450 CYP1A2 metabolizes tizanidine in the liver, which is then excreted through urine and feces [1]. However, ciprofloxacin is a fluoroquinolone antibiotic that inhibits DNA-gyrase in bacteria destroying the DNA. It also acts as an inhibitor of cytochrome P450 CYP1A2, which therefore causes an increase in tizanidine levels in the human body leading to multiple side effects like fatigue, hypotension, bradycardia, and dizziness [1]. Therefore, a formal contraindication exists when tizanidine is co-administered with CYP1A2 inhibitors such as fluoroquinolone antibiotics like ciprofloxacin [1].

## Case presentation

**Figure 1:**
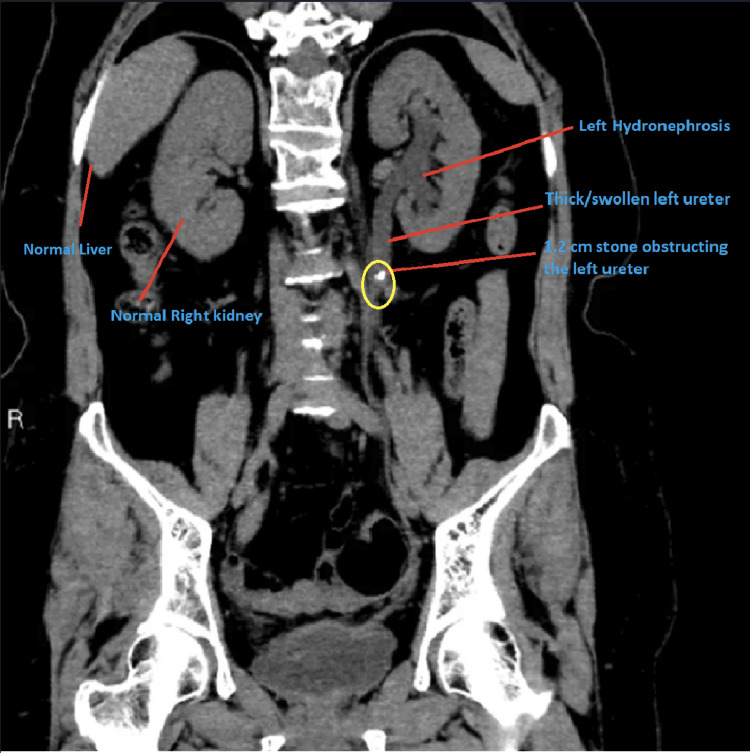
Left ureteral stone. Left ureteral stone causing swollen left ureter along with moderate hydronephrosis of the left kidney.

The patient is a 48-year-old female with a past medical history of lower back pain due to obesity for which she takes 4 mg of tizanidine orally daily along with ibuprofen 200 mg orally as needed. She presents to the hospital with a chief complaint of left flank pain, nausea, and vomiting that has been going on for the past 24 hours. The patient had a CT scan of the abdomen and pelvis performed, which showed a 1.2 cm stone obstructing the left ureter as seen in Figure 1.

The patient was also found to have a urinary tract infection (UTI) and also elevated white blood cell count. The details of the vital signs and laboratory findings are listed in Tables 1, 2. The patient was admitted to the hospital with a diagnosis of ureterolithiasis and sepsis. The patient’s urine was collected for urine culture, and she was started on IV ciprofloxacin 400 mg twice daily along with IV morphine 2 mg every 4 hours as needed for pain. The patient received a normal saline bolus initially at 30 mL/kg/h and then was also started on maintenance normal saline 100 mL/h IV. Urology was consulted, and the patient was taken to the operating room, where the uretic stone was removed, and a JJ stent was placed. The patient tolerated the procedure well. The patient's lab works improved; the white blood cell count went down to normal. The patient's nausea and vomiting completely resolved, and she started tolerating the diet. The patient stayed in the hospital for 48 hours after the procedure was completed and was ready to be discharged the next day as we were still pending urine culture results. The patient's ciprofloxacin was switched from IV to oral. However, the patient requested that she be put back on her home medication, tizanidine, for her lower back pain. The patient was offered acetaminophen and other medications but she declined and wanted to be placed back on her home medication tizanidine. As per the patient's request, tizanidine 4 mg orally was started, and it was administered along with her ciprofloxacin antibiotic. Even though an obvious interaction does exist between the two medications, tizanidine was still continued as interactions are not very common in the clinical setting. However, 2 hours after the medication was administered the patient had profound hypotension, bradycardia, and decreased oxygen saturation. Rapid response was called, and the patient was transferred to the ICU. Table 1 shows the vital signs of the patient during her hospital stay.

**Table 1:** Vital signs

	Normal Range	Hospital Stay Day 1	Hospital Stay Day 2	Hospital Stay Day 3	Hospital Stay Day 4
Temperature (Fahrenheit or °F)	97-98.9	101.4 (high)	98.6	96.8 (low)	98.2
Pulse	60-100	102	86	110	86
Blood pressure (mmHg)	Systolic: 110-139, diastolic: 60-89	106/72 (low)	118/76	62/50 (low)	118/76
O_2_ delivery method	Room air	Room air	Room air	Room air	Room air

Table 2 shows the lab values of the patient during her hospital stay.

**Table 2:** Lab values of the patient during her hospital stay

	Normal Range	Hospital Stay Day 1	Hospital Stay Day 2	Hospital Stay Day 3 - (ICU Day 1)	Hospital Stay Day 4
White blood cells (× 10^9^/L)	4.5-11.0	12.4 (high)	9.40	7.52	8.62
Hemoglobin (g/dL)	13.2-16.6	14.5	13.5	13.2 (low)	14.1
Platelet count (× 10^9^/L)	150-400	225	270	280	275
Comprehensive metabolic panel	Normal	Normal			
Urine culture	Negative	Klebsiella			
Urine drug screen	Normal			Opiates	
Blood culture X2	Normal	Normal			

In the ICU, the patient was started on broad-spectrum antibiotics, and tizanidine was discontinued. She was also started on a levophed drip to help with hypotension but was quickly weaned off within 24 hours. The patient had a urine drug screen performed which was only positive for opiates, likely secondary to her taking morphine in the hospital for pain. The urine drug screen was otherwise negative. The patient had an echocardiogram performed which was normal; carotid ultrasound was normal; a CT head without contrast was also performed, which was normal as well. The telemetry monitor did not show any arrhythmias. The patient had a repeat blood test performed, including lactic acid, which was completely normal. The patient's urine culture grew *Klebsiella*, which was pan-sensitive. The patient was then transferred from the ICU floor to the medsurg floor. She spent less than 24 hours in the ICU. The patient was monitored for 24 more hours and then was later discharged home with Augmentin 875 twice daily for 14 days with a urology follow-up and primary care practitioner follow-up within the next two weeks.

## Discussion

Tizanidine is a common short-acting muscle relaxant used to treat muscle spasms and chronic muscle spasticity in patients. Tizanidine is an alpha-2 adrenergic agonist, which causes a decrease in muscle spasticity and causes muscle relaxation. Tizanidine's main side effects are also derived from its mechanism of action, i.e., alpha-2 agonist properties, which can cause hypothermia, somnolence, hypotension, dizziness, and bradycardia. Tizanidine's half-life is approximately 3 hours. Tizanidine is metabolized via the cytochrome P450 CYP1A2 pathway in the liver [1,2].

Ciprofloxacin is an antibiotic that belongs to the class of fluoroquinolones and is considered a CYP1A2 inhibitor. Therefore, concomitant use of tizanidine with ciprofloxacin should be avoided because it has been shown that ciprofloxacin increases the plasma concentration of tizanidine by 5-20 times, resulting in severe toxicity of tizanidine in the patient [2,3]. Administration of ciprofloxacin along with tizanidine increases the risk of hypothermia, somnolence, hypotension, dizziness, and bradycardia in the patient, which can lead to serious side effects and may even be fatal [4].

Because of these side effects, the tizanidine and ciprofloxacin combination is contraindicated. Even though both these drugs are contraindicated, we still see them being coadministered together in both the inpatient and outpatient settings. One possible reason for their coadministration is the overlap in the indication field. For example, muscle spasticity and injury are frequently treated with tizanidine; however, UTIs and other bladder infections are usually treated early with fluoroquinolones like ciprofloxacin [5].

## Conclusions

Results from multiple studies show that despite the presence of formal contraindication between tizanidine and ciprofloxacin, these drugs are still being prescribed together in clinical practice. Ciprofloxacin is a cytochrome P450 CYP1A2 inhibitor; therefore, when ciprofloxacin is given with tizanidine, it causes a high concentration of tizanidine in the human body because tizanidine is metabolized mainly by cytochrome P450 CYP1A2. A high concentration of tizanidine can cause adverse effects like fatigue, hypotension, and bradycardia. Therefore, the concomitant use of ciprofloxacin with tizanidine should be avoided. More education is needed regarding this drug interaction to avoid this known drug interaction.
